# Mouse Genome Database (MGD)-2018: knowledgebase for the laboratory mouse

**DOI:** 10.1093/nar/gkx1006

**Published:** 2017-10-30

**Authors:** Cynthia L Smith, Judith A Blake, James A Kadin, Joel E Richardson, Carol J Bult, the Mouse Genome Database Group

**Affiliations:** The Jackson Laboratory, 600 Main Street, Bar Harbor, ME 04609, USA

## Abstract

The Mouse Genome Database (MGD; http://www.informatics.jax.org) is the key community mouse database which supports basic, translational and computational research by providing integrated data on the genetics, genomics, and biology of the laboratory mouse. MGD serves as the source for biological reference data sets related to mouse genes, gene functions, phenotypes and disease models with an increasing emphasis on the association of these data to human biology and disease. We report here on recent enhancements to this resource, including improved access to mouse disease model and human phenotype data and enhanced relationships of mouse models to human disease.

## INTRODUCTION

The laboratory mouse is used extensively as a model for investigating the etiopathogenesis of human disease. The relevance of laboratory mouse to biomedical research includes its extensive experimental genetics capabilities, fully-sequenced inbred strain genomes, published genotype to phenotype associations, and data for genome wide coverage of induced variation from mouse large-scale mutagenesis programs (1–4) and reviewed in (5). Resources such as the Collaborative Cross (6) and Diversity Outbred projects (7) capture most of the genetic variation present in laboratory mouse strains and serve as unique platforms for studies of human relevant quantitative traits and complex inherited syndromes. Furthermore, the International Mouse Phenotyping Consortium (IMPC) is generating a catalogue of gene function through systematic generation and phenotyping of a genome-wide collection of traditional gene knockout (KO) and CRIPSR mutant lines in the mouse (8).

The Mouse Genome Database (MGD) is the primary community knowledgebase for mouse phenotype and gene function and mouse models of human disease (9). MGD’s goal is to advance understanding of human biology and disease by facilitating access to integrated genetics and genomic data for the laboratory mouse. To this end, MGD serves as an authoritative resource for the catalog of mouse genes and genome features connecting reference genomic sequence information to mouse biological data, including (i) molecular function, biological process and cellular location information encoded using the Gene Ontology (GO); (ii) a comprehensive listing of mouse mutations, variants and human disease models, with mutant genotypes annotated to Mammalian Phenotype (MP) terms and Disease Ontology (DO) terms and (iii) provide authoritative nomenclature and identifiers for mouse gene names, symbols, alleles and strains as the recognized primary community resource (Table 1). Standardization of mouse nomenclature and annotation of data with commonly used biological ontologies and standardized vocabularies ensure that data are consistently annotated, making precise data mining possible.

**Table 1.** Data for which MGD serves as an authoritative source. In addition to providing unique IDs and symbols for genes, alleles and strains, MGD expertly curates functional, phenotype and disease model data from literature into MGD

Data type	Description
Unified mouse genome feature catalog	Catalog of integrated predictions from Ensembl, NCBI, Havana/Vega; used by NCBI, IMPC, etc.
Gene Ontology (GO) annotations for mouse	Curated from literature and integrated from others
Mouse phenotype annotations	Curated from literature, integrated with data from large scale projects
Mouse models of human disease	Curated mouse models of human disease annotated with Disease Ontology
Gene to nucleotide sequence association	Co-curation with MGA (Mouse Genome Annotation) group
Gene to protein sequence association	Co-curation with UniProt and Protein Ontology groups
Mammalian Phenotype (MP) Ontology	Developed, distributed and used by MGD; also used by RGD, IMPC, DMDD, etc.
Gene and genome feature symbols, names and IDs	Created using International Nomenclature Guidelines in coordination with human and rat nomenclature groups
Mutation symbols, names and IDs	Nomenclature and IDs for mouse mutations are assigned and provided by MGD
Mouse strain nomenclature and IDs	Created and provided by MGD; nomenclature assistance is also provided to other mouse repositories

MGD is a core component of the Mouse Genome Informatics (MGI) consortium (http://www.informatics.jax.org). Other database resources coordinated through the MGI consortium include the Gene Expression Database (GXD) (10), the Mouse Tumor Biology Database (MTB) (11), the Gene Ontology project (GO) (12), MouseMine (13), the International Mouse Strain Resource (IMSR) (14) and the CrePortal database of recombinase expressing mice (www.CrePortal.org, unpublished). Data and information for these resources are obtained through a combination of expert curation of the biomedical literature and by automated or semi-automatic processing of data sets downloaded from more than fifty other data resources. Metrics of current MGD content is shown in Table 2.

**Table 2.** Summary of MGD content, September 2017

Genes and genome features with nucleotide sequence data	47 693
Genes with protein sequence data	24 317
Genes with human orthologs	17 089
Genes with rat orthologs	18 509
Genes with GO annotations	24 502
Total GO annotations	312 109
Mutant alleles in mice	51 378
Genes with mutant alleles in mice	12 401
Mouse QTL	6257
Genotypes with phenotype annotation (MP)	60 951
Total MP annotations	315 657
Mouse models (genotypes) associated with human diseases	6027
References in the MGD bibliography	237 578

In this report, we highlight several improvements to the capture, annotation, integration and presentation of data associated with mouse models of human disease. These include the incorporation of the Disease Ontology (DO), new disease detail pages and ontology browser with listings of associated genes and mouse models, improved ontology vocabulary browsers and incorporation of a Human Phenotype Ontology (HPO) browser. We added human disease to phenotype relationships from Orphanet, which expands our number of human diseases annotated to HPO terms. We now include phenotype data from Deciphering the Mechanisms of Developmental Disorders (DMDD) project. Finally, we are participating with the new Alliance of Genome Resources member groups in efforts to create a new data resource for comparative biology (http://www.alliancegenome.org).

## NEW FEATURES AND IMPROVEMENTS

The three primary areas of improved and new functionality in MGD center on disease and phenotype annotation and the user interfaces used to deliver these annotations to the biomedical research community.

### Disease Ontology (DO) is incorporated into MGD

The Disease Ontology (DO) is a community effort to provide standard terms for annotating phenotypic data (15). It is a hierarchical ontology, built on a Directed Acyclic Graph (DAG) structure, that integrates vocabularies from MeSH, ICD, NCI Thesaurus, SNOMED, UMLS, Orphanet, EFO and OMIM. Its hierarchical structure permits a range of detail from high-level, broadly descriptive terms to low-level, very specific terms. This range is useful for annotating mouse model data to the level of detail known and for searching for this information using either whole systems or specific terms as search criteria.

**Figure 1.**
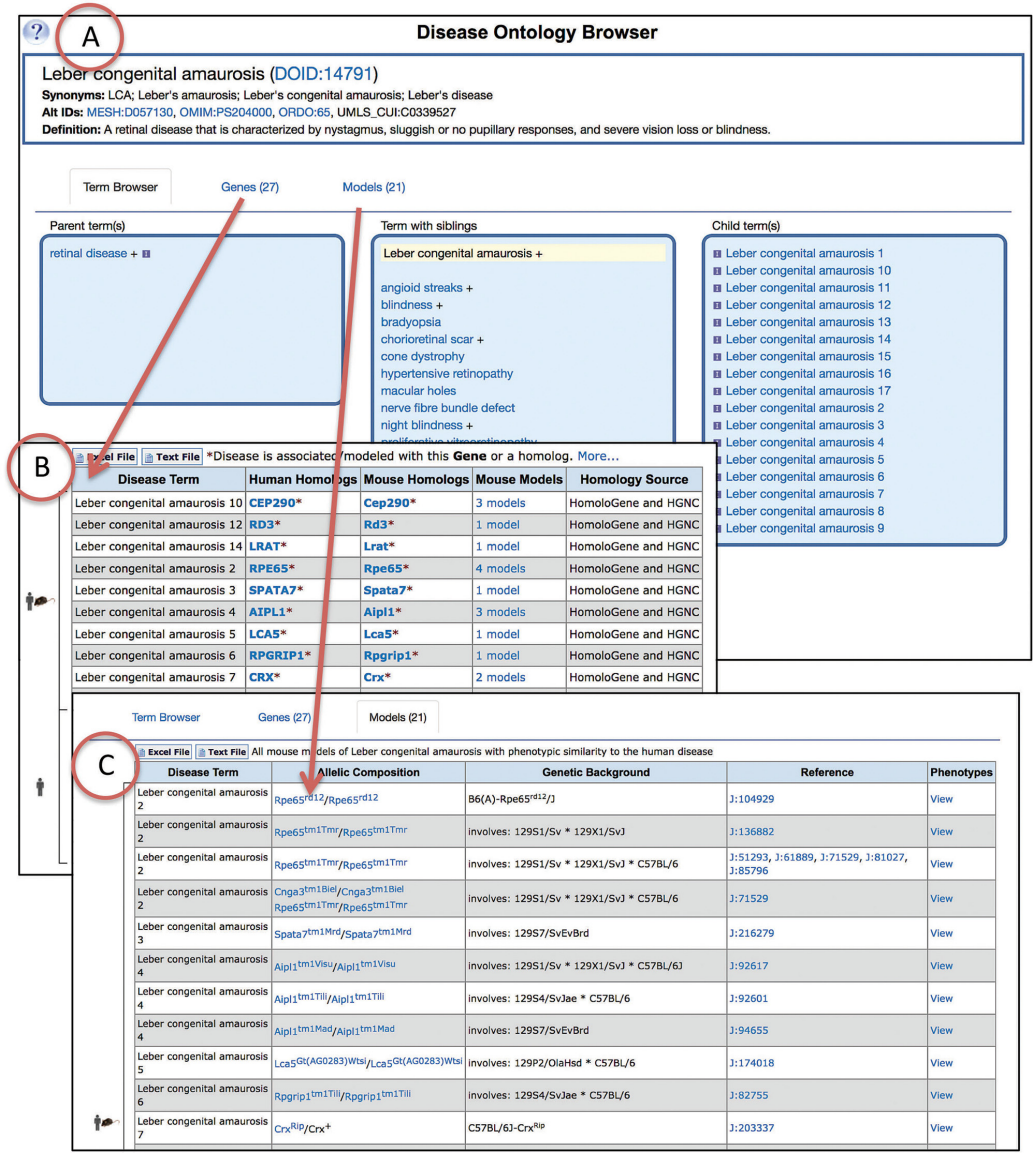
Disease Ontology Browser at Mouse Genome Informatics. Example of a new disease detail page and associated mouse and human data available for Leber congenital amaurosis. (**A**) Shown are the Disease Ontology (DO) term, definition, synonyms and IDs for this DO term. Additional matching terms at other resources are shown and linked when available at another resource. Graphical views of the ontology are also available. (**B**) The genes tab of the browser lists all human and mouse orthologs associated with the disease or subclasses of the disease. Mouse models are listed, if available. (**C**) The models tab lists the disease and genotype of mouse models of the disease along with supporting reference information and links to phenotype data for each model.

We have adopted the use of Disease Ontology in MGD to annotate mouse models of human disease and participated in updating and making new additions to DO. Previously we relied on disease titles from the Online Mendelian Inheritance in Man (OMIM) database (16) for annotations of mouse models of human disease. This approach limited the scope of disease terms because of the focus of OMIM on human diseases with familial inheritance. Furthermore, the OMIM term list does not exist in a complete hierarchical structure, limiting search and retrieval efficiency. Existing MGI mouse-human disease model annotations to OMIM terms have been translated to DO terms. The new MGD Disease Ontology Browser (Figure 1) allows users to navigate the ontology and see associated genes and mouse models, either in a tree or graphical view. Links to other disease vocabularies and ontologies, including OMIM, are provided when available. The additional genes tab and models tab for terms show all data for the selected term and also for any of the more specific subclasses in the ontology. The MGI Quick Search field, Human-Mouse Disease Connection, and other advanced query forms will support searches using the DO terms or IDs.

### Ontology browser redesign

**Figure 2.**
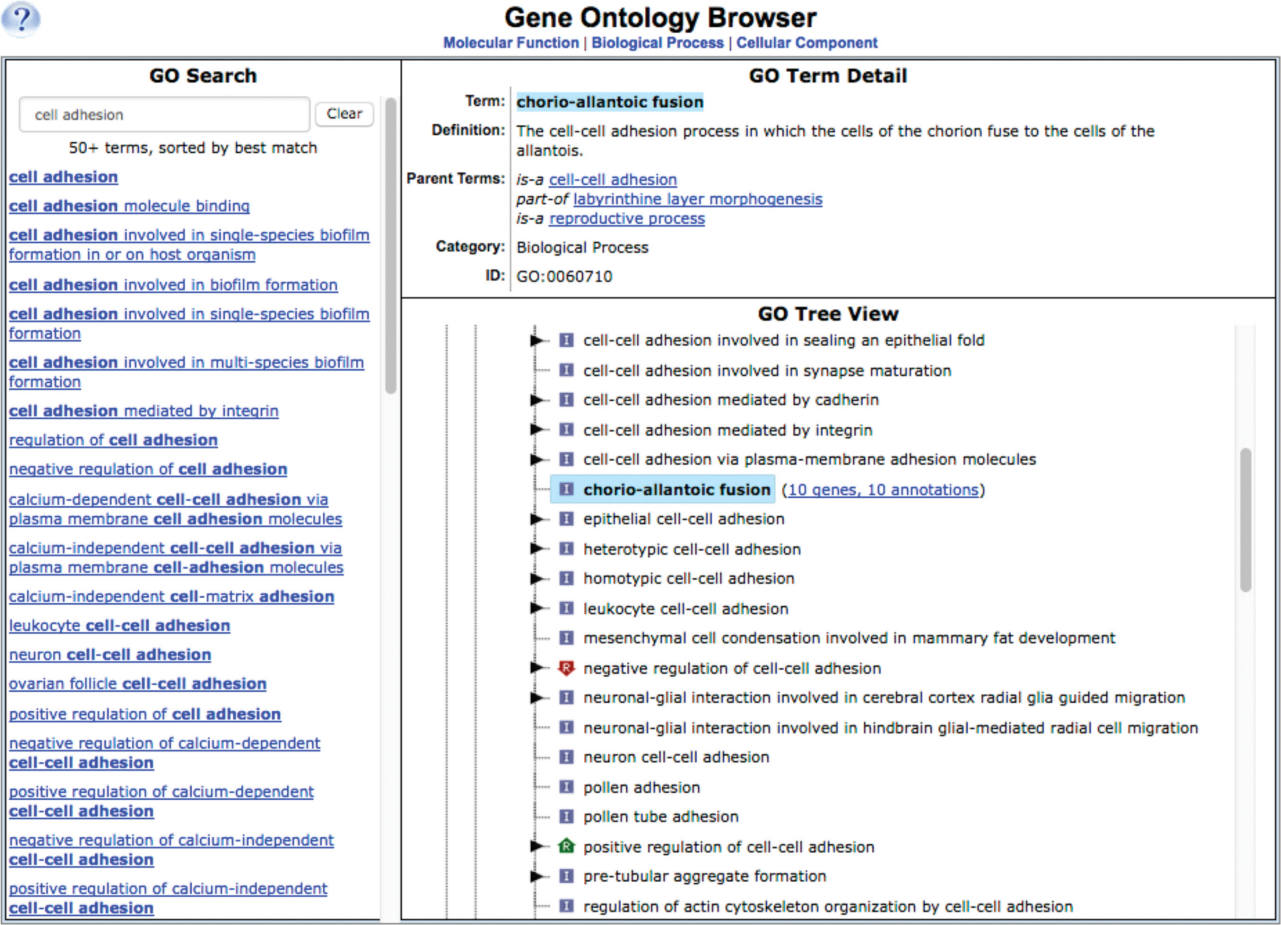
Gene Ontology Browser. The new MGD ontology browsers have an autocomplete search feature and display linked matching terms. Shown here is an example from the Gene Ontology Browser. GO term names, definitions, IDs, superclasses (parent terms) and term relations are listed and terms are shown in a tree view. The example shown here is a search for ‘cell adhesion’ showing all terms containing this text string. The subclass ‘chorio-allantoic fusion’ is selected. Different branches may be viewed by clicking on the different parent terms under the defintion. Each biological term is connected to annotated genes via a link in the tree view following the term name; in this example, ten different genes are annotated to this biological process term. Other terms with subclasses are expanded by clicking on the triangle toggle to the left of the term name.

Improvements to the Mammalian Phenotype (MP), Gene Ontology (GO), and the Adult Mouse Anatomy browsers include implementation of new format and search options including an autocomplete option to assist in finding relevant terms (Figure 2). Tree views include easy navigation with links to superclasses and to annotated mouse phenotype, function and expression data. Toggles in the tree structure allow expansion or collapse of particular sections of interest.

MGD has also implemented a new browser created by MGI that features the Human Phenotype Ontology (17) developed by Peter Robinson and colleagues (http://www.informatics.jax.org/vocab/hp_ontology). Users can either browse or search the HPO browser to view terms, definitions and links to disease detail pages that are associated with the phenotypic feature at the Human-Mouse Disease Connection (HMDC) portal.

### Improvements to the Human-Mouse Disease Connection (HMDC)

The Human-Mouse Disease Connection (HMDC, http://www.diseasemodel.org) is a translational tool designed for exploring and comparing human and mouse phenotypes and their associations with known human diseases. It also provides rapid access to mouse model resources and supporting references. Searches can be initiated based on human or mouse data using one or more parameters, including genes, genomic locations, phenotypes and diseases. New features and data include searches by Disease Ontology terms to group and display disease classes, and the incorporation of human disease and phenotype relationships from Orphanet (http://www.orpha.net). These new data sets add to the existing OMIM disease-to-phenotype data from the HPO project (17) implemented previously.

### Inclusion of Deciphering the Mechanisms of Developmental Disorders (DMDD) data

**Figure 3.**
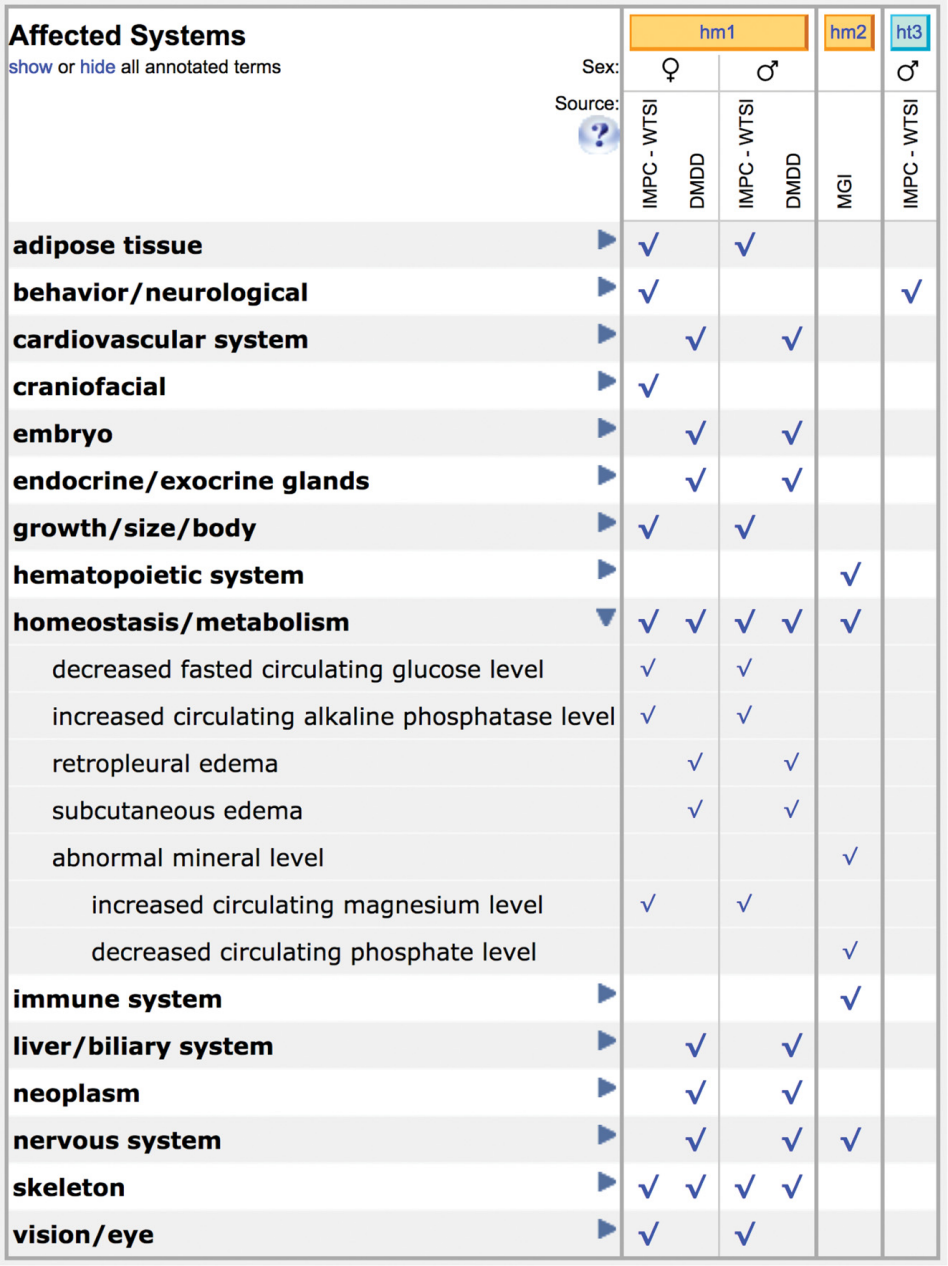
Phenotype systems grid for *Slc20a2^tm1a(EUCOMM)Wtsi^*. Shown is Phenotype systems grid from the allele detail page the for *Slc20a2^tm1a(EUCOMM)Wtsi^* allele featuring DMDD data integrated with IMPC data and published data curated by MGI. Blue triangular toggles to the right of the system name may be opened for comparison of data details across genotypes. Further phenotype details including detailed phenotype terms and references may be accessed by clicking genotype links (e.g. ‘hm1’) or checkmarks on the phenotypes grid. Other included information on the allele detail page for this mutation (not shown) are links to the gene detail page, mutation origin and description, human disease information, links to repositories carrying the mouse mutation and references.

MGD now integrates mouse embryonic mutant phenotype data generated by the Deciphering the Mechanisms of Developmental Disorders (DMDD) project (18). MGD currently has information for 63 mouse lines, and more phenotype data for ∼200 additional lines will be added as they become available. All of the DMDD mouse lines are derived from International Knockout Mouse Consortium (IKMC)’s Knockout Mouse Project (KOMP) or European Conditional Mouse Mutagenesis Program (EUCOMM) ES cells (19) or CRISPR-induced lines (20). Mouse mutations are annotated to MP terms by DMDD and are shown in parallel with other published and submitted data on these mutations. Expression data for these lines are also available from the Gene Expression Database (GXD) at MGI (10). Shown in Figure 3 is an example of DMDD data submitted for the *Slc20a2^tm1a(EUCOMM)Wtsi^* mutation together with data submitted by IMPC and from published literature curated at MGI.

### Other enhancements

In addition to the major enhancements described above, several minor enhancements were implemented. Researchers can now search for genome features that are still on unlocalized and unplaced contigs for the reference genome assembly (GRCm38). MGD data loads from IMPC were modified to load and integrate phenotype data generated from CRISPR mutations in addition to ES cell line knockout mutations, increasing the number of annotated phenotype-genotype data in MGD. MGD also updated the load of GO annotations from the Go consortium site to use the new Gene Product Association Data (GPAD, http://www.geneontology.org/page/gene-product-association-data-gpad-format) annotation file format which supports inclusion of additional metadata in contrast to the previously used Gene Association File (GAF).

### MGD and the Alliance of Genome Resources

MGD is one of the founding members of the Alliance of Genome Resources (AGR), a new data resource integration effort among the major model organism database (MOD) groups and the Gene Ontology Consortium (GOC). The founding members of the AGR are: the Gene Ontology Consortium, Mouse Genome Database (MGD), FlyBase, WormBase, Saccharomyces Genome Database (SGD), Rat Genome Database (RGD), Zebrafish Information Network (ZFIN). The AGR will work to standardize access to and display of common data types from different model organisms to better support comparative biology for biomedical researchers. The formation of the AGR builds on the collaborative activities between the MODs and GO over several years seeking to enhance data integration, exchange, and the use of common data standards. Now these groups will merge key activities and data representations, coordinating data retrieval and analysis within the comparative perspective. The initial release of the public web portal for the AGR (http://www.alliancegenome.org) is scheduled for October 2017. Among the data types to be included in the initial release are gene details such as gene name and symbol, genomic location, orthology, function annotations, and disease associations. Longer-term goals of the Alliance include adding other model organisms, data types, and analysis tools within a common shared infrastructure.

## IMPLEMENTATION AND PUBLIC ACCESS

The primary MGI database (‘production’) is a highly normalized relational database designed and optimized for data integration and incremental updating. This database is the locus of data loads and ongoing expert data curation. It resides in a PostgreSQL server behind a firewall and is not accessible by the public. In contrast, our public web interface is backed by a combination of a highly unnormalized databases (also in PostgreSQL) and Solr/Lucene indexes, designed for high performance query and display in a read-only environment. The front end data stores are refreshed from the production master on a weekly basis. The separation between the public and production (private) architectures provides a large measure of flexibility in project planning, as either side can (and often does) change without affecting the other.

MGD broadcasts data in a variety of ways to support basic research communities, clinical researchers and advanced users interested in programmatic or bulk access. MGD provides free public web access to data from http://www.informatics.jax.org. The web interface provides a simple ‘Quick Search’, available from all web pages in the system and is the most used entry point for users. Various advanced query forms are provided to support precise parameter searching. Data may be retrieved from most results pages by downloading text or Excel files, or forwarding results to Batch Query or MouseMine analysis tools (see below).

MGD offers batch querying interfaces for data retrieval for users wishing to retrieve data in bulk. The Batch Query tool (http://www.informatics.jax.org/batch) (21) is used for retrieving bulk data about lists of genome features. Feature identifiers can be typed in or uploaded from a file. Gene IDs from MGI, NCBI GENE, Ensembl, VEGA, UniProt and other resources can be used. Users can choose the information set they wish to retrieve, such as genome location, GO annotations, list of mutant alleles, MP annotations, Reference SNP IDs or Disease Ontology (DO) terms. Results are returned as a web display, or in tab delimited text or Excel format. Results may also be forwarded to MouseMine (see below).

MGD data access is available through MouseMine (http://www.mousemine.org) (13), an instance of InterMine that offers flexible querying, templates, iterative querying of results and linking to other model organism InterMine instances. MouseMine access is also available via a RESTful API, with client libraries in Perl, Python, Ruby, Java and JavaScript.

This year, MGD retired its FTP server and other back end upgrades. The files that were accessible via FTP are now downloadable from http://www.informatics.jax.org/downloads/. MGD provides a large set of regularly updated database reports from this site. Direct SQL access to a read-only copy of the database is also offered (contact MGI user support for an account). MGI User Support is also available to assist users in generating customized reports on request.

## OUTREACH

MGD User Support staff are available for on-site help and training on the use of MGD and other MGI data resources as well as providing off-site workshop/tutorial programs (roadshows) that include lectures, demos and hands-on tutorials. To inquire about hosting an MGD roadshow, email mgi-help@jax.org.

Members of the MGD User Support team can be contacted via email, web requests, phone or fax.

- World wide web: http://www.informatics.jax.org/mgihome/support/mgi_inbox.shtml
- Facebook: https://www.facebook.com/mgi.informatics
- Twitter: https://twitter.com/mgi_mouse and https://twitter.com/hmdc_mgi
- Email access: mgi-help@jax.org
- Telephone access: +1 207 288 6445.
- Fax access: +1 207 288 6830.

## CITING MGD

For a general citation of the MGI resource, researchers should cite this article. In addition, the following citation format is suggested when referring to datasets specific to the MGD component of MGI: mouse genome database (MGD), MGI, The Jackson Laboratory, Bar Harbor, Maine (URL: http://www.informatics.jax.org). Type in date (month, year) when you retrieved the data cited.

## MOUSE GENOME DATABASE GROUP

A. Anagnostopoulos, A. Andrews, R.M. Baldarelli, J.S. Beal, S.M. Bello, O. Blodgett, N.E. Butler, K. Christie, L.E. Corbani, H.J. Drabkin, R. Espinoza, J. Franco, S.L. Giannatto, P. Hale, D.P. Hill, L. Hutchins, M. Law, J.R. Lewis, M. McAndrews, N. Mez, D. Miers, H. Motenko, L. Ni, H. Onda, M. Perry, J.M. Recla, D.J. Reed, B. Richards-Smith, D. Sitnikov, M. Tomczuk, L. Wilming and Y. Zhu.
